# Effects of short-term exposure to head-down tilt on cerebral hemodynamics: a prospective evaluation of a spaceflight analog using phase-contrast MRI

**DOI:** 10.1152/japplphysiol.00841.2015

**Published:** 2016-03-24

**Authors:** Karina Marshall-Goebel, Khalid Ambarki, Anders Eklund, Jan Malm, Edwin Mulder, Darius Gerlach, Eric Bershad, Jörn Rittweger

**Affiliations:** ^1^Institute of Aerospace Medicine, German Aerospace Center (DLR), Cologne, Germany;; ^2^Faculty of Medicine, University of Cologne, Cologne, Germany;; ^3^Department of Radiation Sciences, Umeå University, Umeå, Sweden;; ^4^Centre of Biomedical Engineering and Physics, Umeå University, Umeå, Sweden;; ^5^Department of Pharmacology and Clinical Neuroscience, Umeå University, Umeå, Sweden;; ^6^Department of Neurology, Baylor College of Medicine, Houston, Texas; and; ^7^Department of Pediatric and Adolescent Medicine, University of Cologne, Cologne, Germany

**Keywords:** visual impairment and intracranial pressure, microgravity, cerebral blood flow, head-down tilt, MRI

## Abstract

Alterations in cerebral hemodynamics in microgravity are hypothesized to occur during spaceflight and could be linked to the Visual Impairment and Intracranial Pressure syndrome. Head-down tilt (HDT) is frequently used as a ground-based analog to simulate cephalad fluid shifts in microgravity; however, its effects on cerebral hemodynamics have not been well studied with MRI techniques. Here, we evaluate the effects of *1*) various HDT angles on cerebral arterial and venous hemodynamics; and *2*) exposure to 1% CO_2_ during an intermediate HDT angle (−12°) as an additional space-related environmental factor. Blood flow, cross-sectional area (CSA), and blood flow velocity were measured with phase-contrast MRI in the internal jugular veins, as well as the vertebral and internal carotid arteries. Nine healthy male subjects were measured at baseline (supine, 0°) and after 4.5 h of HDT at −6°, −12° (with and without 1% CO_2_), and −18°. We found a decrease in total arterial blood flow from baseline during all angles of HDT. On the venous side, CSA increased with HDT, and outflow decreased during −12° HDT (*P* = 0.039). Moreover, the addition of 1% CO_2_ to −12° HDT caused an increase in total arterial blood flow (*P* = 0.016) and jugular venous outflow (*P* < 0.001) compared with −12° HDT with ambient atmosphere. Overall, the results indicate decreased cerebral blood flow during HDT, which may have implications for microgravity-induced cerebral hemodynamic changes.

## Abstract (precis)

*This is the first study to examine cerebral hemodynamics using phase-contrast MRI during various angles of head-down tilt. Furthermore, the study investigated the additional effects of increased ambient carbon dioxide during head-down tilt as an analog to the environment onboard the International Space Station*.

## NEW & NOTEWORTHY

*This is the first study to examine cerebral hemodynamics using phase-contrast MRI during various angles of head-down tilt. Furthermore, the study investigated the additional effects of increased ambient carbon dioxide during head-down tilt as an analog to the environment onboard the International Space Station*.

the cerebral hemodynamic system is closely regulated under a variety of physiological conditions to maintain brain homeostasis. In space, gravity-induced hydrostatic pressure gradients normally present on Earth vanish, and a 2-liter headward fluid shift occurs (24). It is hypothesized that this cephalad fluid shift may increase intracranial pressure (ICP) due to impaired cerebral venous drainage (23). Given that cerebral venous outflow is directly related to cerebrospinal fluid outflow, this may lead to some of the underlying structural and functional ophthalmic changes seen in astronauts with the Visual Impairment and Intracranial Pressure (VIIP) syndrome, including optic disk edema, globe flattening, and hyperopic shifts (19, 21).

Head-down tilt (HDT) bed rest has been used for decades as a ground-based spaceflight analog to simulate the physiological effects of microgravity on fluid redistribution (27, 34). However, long-duration HDT bed-rest studies at −6° have failed to reproduce ophthalmic findings similar to those seen in the VIIP syndrome (33). This raises the question as to whether the −6° HDT angle creates a sufficient hydrostatic pressure gradient to induce cephalad fluid shifting as in microgravity. Therefore, the presented study examined the effects of more extreme tilt angles (−12° and −18° HDT). In addition, ambient carbon dioxide (CO_2_) levels on the International Space Station (ISS) are about 10 times higher than terrestrial levels (on average 0.45 vs. 0.04%) (20). As a potent cerebral arteriolar vasodilator, exposure to elevated ambient CO_2_ levels increases cerebral blood flow (CBF), which may increase intracranial blood volume and, therefore, pressure. While transient fluctuations in ICP are not pathological in nature and may actually assist with CBF regulation, a sustained increase in mean ICP may be a major contributor to the ophthalmic changes associated with the VIIP syndrome.

In this study, we hypothesized that HDT posture would result in a disturbance in cerebral circulation, leading to decreased venous outflow and arterial inflow. Thus our study aim was to evaluate the effects of *1*) various degrees of HDT (−6°, −12°, and −18°) on the major cerebral/neck veins and arteries using phase-contrast magnetic resonance imaging (PC-MRI); and *2*) exposure to increased ambient CO_2_ in combination with an intermediate HDT angle (−12°) as an additional analog to the ISS environment.

## MATERIALS AND METHODS

### 

#### Study design.

Nine healthy male subjects (mean ± SD age: 25 ± 2.4 yr; mean ± SD height: 183 ± 6 cm; mean ± SD body mass index: 24.1 ± 2.4 kg/m^2^) participated in the study. All test subjects underwent a medical screening before inclusion in the study, which included an oral interview about medical history and current health status, physical examination, urine and blood samples, electrocardiogram, spirometry test, and eye examination. Criteria for inclusion in the study included no prescription medications, nonsmokers, and a body mass index between 19 and 30 kg/m^2^. Exclusion criteria included history of increased ICP, neurological or cardiovascular diseases, and any significant pathology seen on MRI.

The study was divided into four experimental conditions: −6° HDT, −12° HDT, −18° HDT, and −12° HDT, with 1% CO_2_ atmosphere. During each session, the subjects were placed in the supine (0°) position for 3 h, followed by one of the four experimental condition positions, randomly selected to reduce order-related effects, for a total of 5 h (PC-MRI scans started at 4.5 h HDT). Additionally, there were at least 5 days between sessions to prevent carry-over effects of the previous condition. Nutritional and fluid intake was standardized during the experiments: in the 24 h preceding each experiment, subjects were instructed to consume 40 ml of fluid/kg body wt and to refrain from drinking caffeinated drinks. On the days of the experiments, subjects were provided with an individualized, habitual, and thereafter standardized breakfast with 250 ml of water and 200 ml of juice. After baseline data collection and immediately preceding HDT, subjects consumed 250 ml of water and a standardized snack. After 2 h in the HDT position, subjects received 200 ml of a high-caloric shake. Urination breaks were also scheduled at predetermined time points, including just before entry into the MRI scanner. The study was conducted in accordance with the ethics principles stated in the Declaration of Helsinki and approved by the ethics commission of the regional medical board (Ärztekammer Nordrhein). Written, informed consent was obtained from all volunteers before the start of the study. The study was carried out in the Envihab facility at the German Aerospace Center (DLR), and the presented results represent a subset of data collected from a larger study evaluating various aspects of human physiological responses to short-term HDT.

#### Magnetic resonance imaging.

**Fig. 1.**
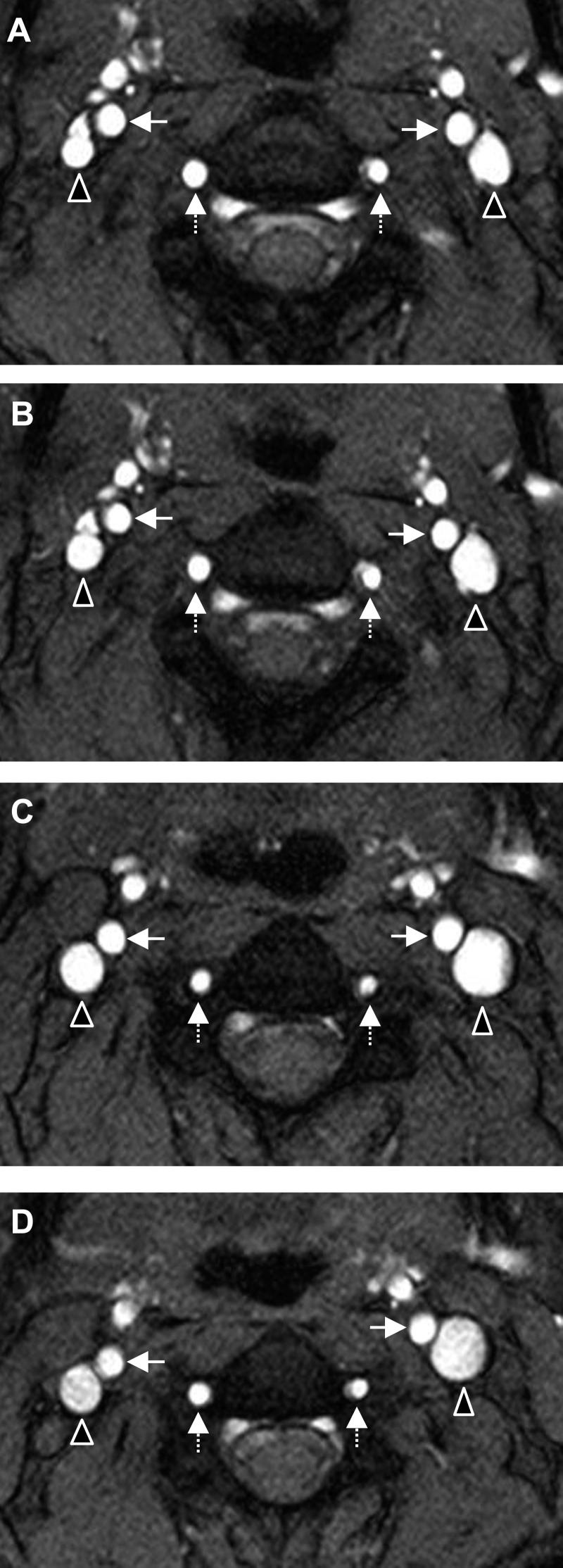
Cross-sectional magnitude images from a phase-contrast MRI sequence taken between the second and third vertebrae in one subject. The images show the left and right internal jugular veins (triangles), internal carotid arteries (solid arrows), and vertebral arteries (dashed arrows) at 0° baseline (*A*), −6° HDT (*B*), −12° HDT (*C*), and −18° HDT (*D*).

MRI data were acquired using a Biograph mMR 3-Tesla scanner (Siemens, Erlangen, Germany) with a 16-channel head-neck coil. All baseline scans were taken in the supine position on the equipped standard, horizontal bed. To maintain the HDT positions for the entire session, specially designed wedges were developed for each condition (−6°, −12°, and −18° HDT). In addition, during the −12° HDT with 1% CO_2_, the atmosphere was maintained during the MRI scans using a mask and tank system (tank composition: 1% CO_2_, 20% O_2_, N_2_ balance). The duration of the MRI examination was ∼1 h, with the PC-MRI scans beginning ∼30 min into the MRI exam. A 3D T1-weighted MPRAGE (magnetization-prepared rapid acquisition of gradient echo) sequence was used for planning a perpendicular PC-MRI plane above the bifurcation of the carotid artery. The blood flow measurements were obtained for the bilateral internal carotid arteries (ICA), vertebral arteries (VA), and internal jugular veins (IJV) between the second and third cervical vertebrae. Figure 1 shows an example of a cross-sectional magnitude image from one subject during baseline (Fig. 1*A*) and at −6° (Fig. 1*B*), −12° (Fig. 1*C*), and −18° HDT (Fig. 1*D*).

Blood flow rate was acquired using PC-MRI with the following parameters: total acquisition time, 7-11 min [heart rate (HR) dependent]; repetition time, 21.4 ms; echo time, 6.71 ms; slice thickness, 5 mm; 15° flip angle; 200 × 200 mm field of view; 320 × 320 acquisition matrix; 0.625 × 0.625 mm in-plane resolution with 32 calculated phases; and 2 averages. Parallel imaging (iPAT) with a GRAPPA algorithm acceleration factor 2 was used with 24 reference lines. The velocity encoding (VENC) value was 70 cm/s, and retrospective peripheral pulse gating was used.

#### PC-MRI image analysis.

Postprocessing of the PC-MRI data was performed using the Segment v1.8 software (http://medviso.com/products/segment/) by an experienced scientist. Using the magnitude images, a region of interest was manually drawn to delineate the arterial or venous lumen. A fully automatic contour-active model was used to track the edge of the lumen during the cardiac cycle (13). The program follows the lumen boundaries as the vessel changes shape and becomes more spherical with increased filling. Blood flow rate (ml/min) in each vessel was automatically computed by multiplying the mean velocity (cm/s) by the mean cross-sectional area (CSA) (cm^2^) during the cardiac cycle. This cardiac cycle represents the average of many cardiac cycles taken over 7–11 min.

Total arterial inflow was defined as the sum of the mean flow rates of the bilateral ICAs and VAs, and total jugular venous outflow was defined as the sum of the mean flow rates of the bilateral IJVs. Total arterial CSA was calculated as the sum of the CSAs of the bilateral ICAs and VAs, and total jugular venous CSA was calculated as the sum of the CSAs of the bilateral IJVs.

#### Cardiovascular variables.

Both during baseline and after 4-h HDT (before entry into the MRI scanner), continuous beat-by-beat arterial finger blood pressure was measured with a Finometer (Finapres Medical Systems, Amsterdam, The Netherlands) for 10 min. Subjects also had a three-lead electrocardiogram to obtain beat-by-beat HR (Biopac Systems, Goleta, CA).

#### Respiratory variables.

End-tidal CO_2_ (ET_CO_2__) and minute ventilation were measured for 10 min at baseline and after 3.5-h HDT with the Innocor system (Innovision, Odense, Denmark). The last 5 min of recordings were used for analysis.

#### Statistical analyses.

**Fig. 2.**
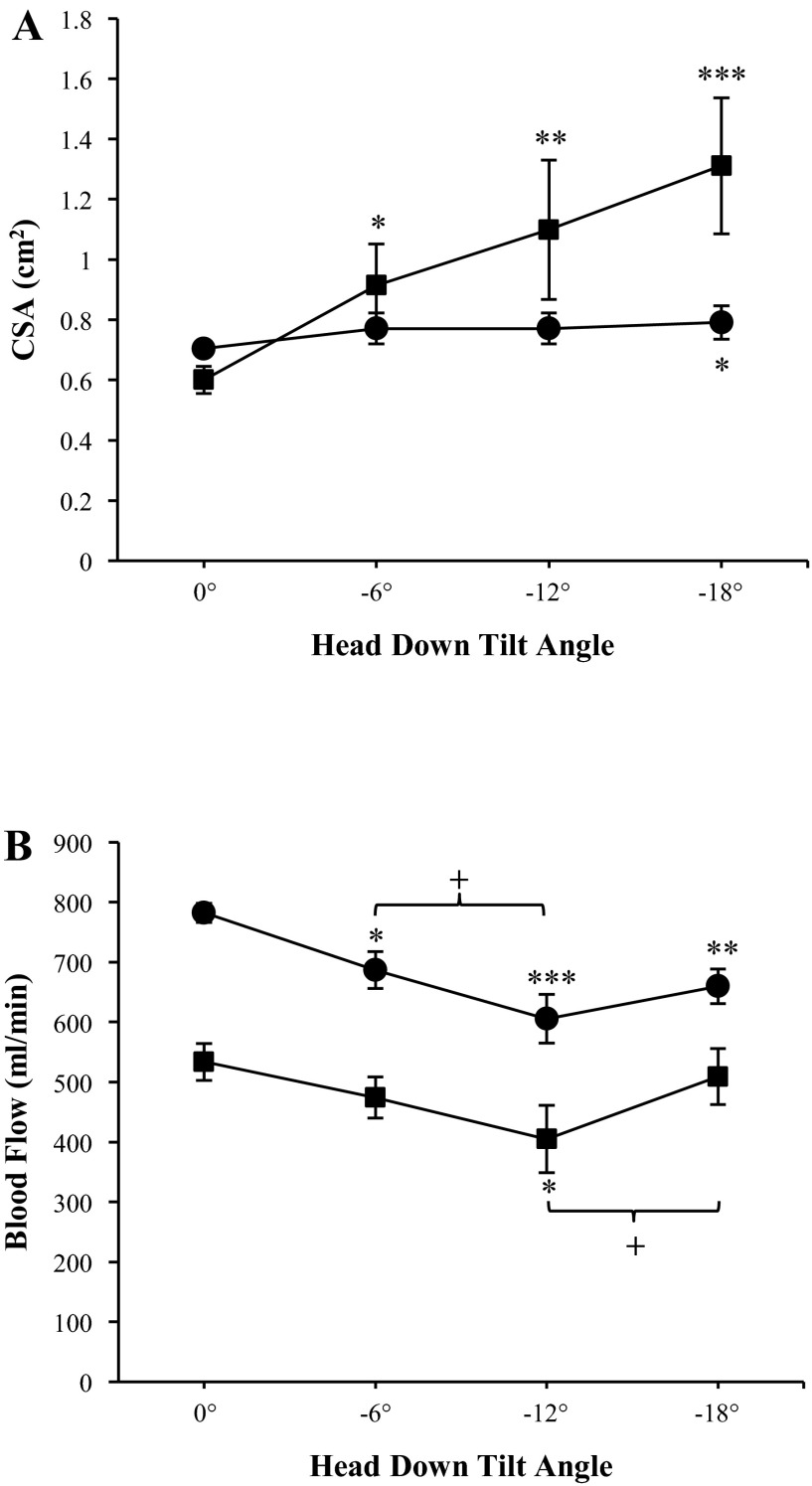
Effects of different head-down tilt angles on total cross-sectional area (CSA; *A*) and total flow (*B*) for the arterial (●) and internal jugular venous (■) systems. Measurements were taken at baseline (0°) and after 4.5-h head-down tilt at various angles with phase-contrast MRI. Flow was calculated by multiplying blood flow velocity by the CSA for each vessel. Blood flow through the bilateral internal carotid and vertebral arteries were summed to give total arterial inflow, and blood flow through the bilateral internal jugular veins were summed to give jugular venous outflow. Values are means ± SE. *P <0.05. **P <0.01. ***P <0.001. ^+^*P* <0.05.

**Fig. 3.**
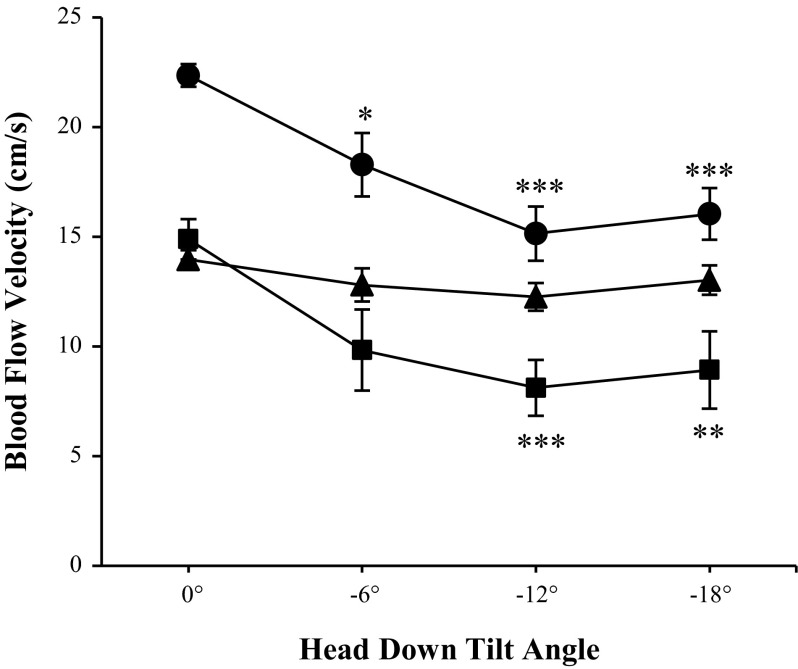
Blood flow velocity for the internal carotid arteries (●), vertebral arteries (▲), and internal jugular veins (■) at various head-down tilt angles. Measurements were taken at baseline (0°) and after 4.5-h head-down tilt with phase-contrast MRI. Values are means ± SE. *P < 0.05. **P < 0.01. ***P < 0.001.

**Fig. 4.**
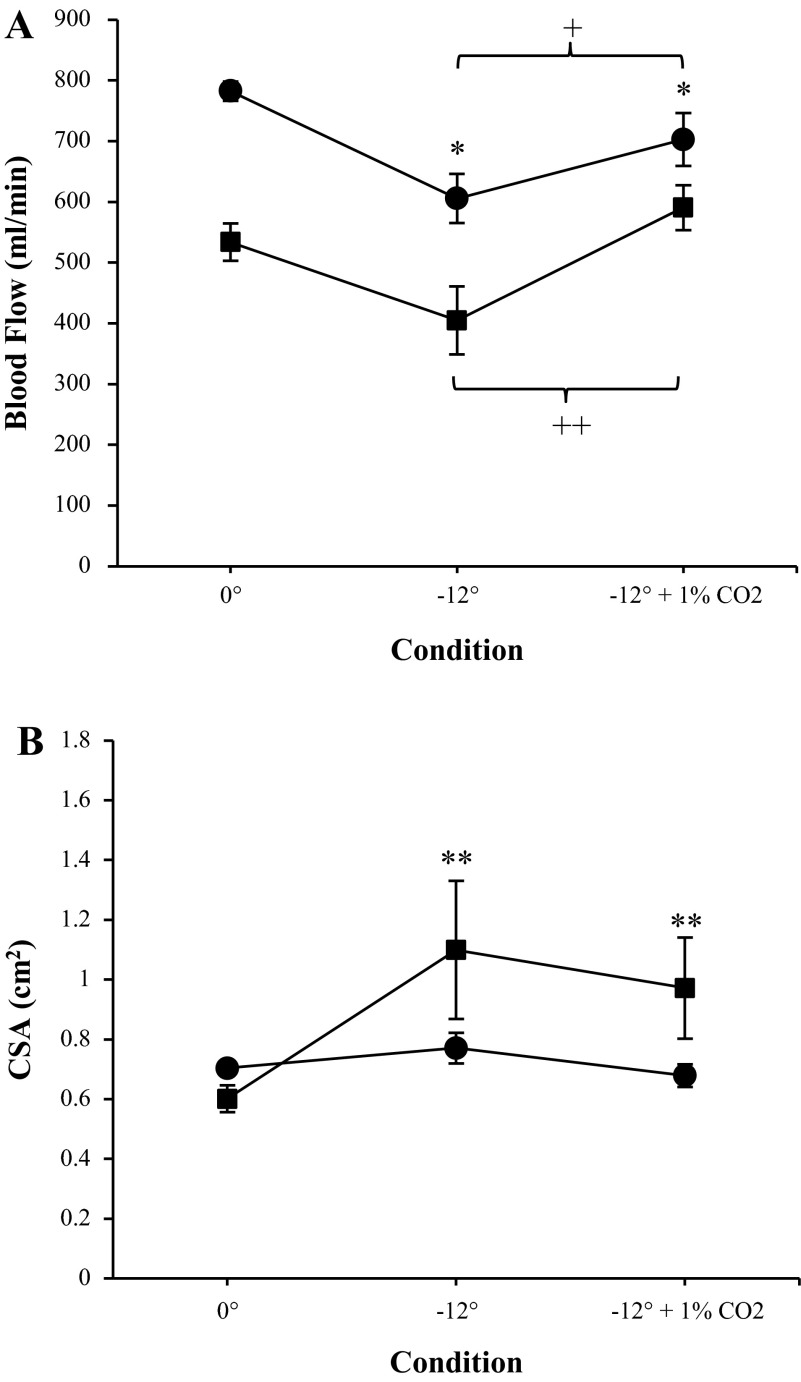
Effects of −12° head-down tilt with and without a 1% CO_2_ atmosphere on phase-contrast MRI-derived total arterial inflow (●; A) and internal jugular venous outflow (■; *A*) and total arterial CSA (●) and internal jugular venous CSA (■; *B*). Measurements were taken at baseline (0°) and after 4.5-h head-down tilt. Flow was calculated by multiplying blood flow velocity by the CSA for each vessel. Flow through the left and right internal carotid arteries and vertebral arteries were summed to give total arterial inflow. Flow through the left and right jugular veins were summed to give venous outflow. Values are means ± SE. *P < 0.05. **P < 0.01. ^+^*P* < 0.05. ^++^*P* < 0.01.

ANOVA and linear mixed effect models with time and condition (−6°, −12°, −18°, −12° + 1% CO_2_) as main effects and subject ID as random effect were constructed to assess intervention effects. When the vessels were examined separately, side was included as an additional term, allowing two-way interaction with condition and time. Variances were allowed to differ between participants and intervention, and linear mixed-effect models were optimized according to Akaike's information criterion. Bonferroni contrast testing was used to compare HDT conditions to baseline supine and to compare between different angles. All baselines were lumped together for figures. Flow values between the bilateral ICAs, VAs, and IJVs were compared with detect lateral dominance. Statistical analyses were carried out using the R-environment in its version 3.1.2, 64-bit (www.r-project.org) and IBM SPSS Statistics Version 20 (IBM, Armonk, NY). Data are shown as means and standard errors (SE) in Figs. 2–4 to demonstrate uncertainty and given as means and standard deviations (SD) in the text and Table 1. The level for statistical significance was set to α = 0.05 and β was set to 0.2.

## RESULTS

### 

#### CSA and blood flow velocity.

PC-MRI-derived measurements of CSA during HDT are shown in Fig. 2*A*. Total jugular venous CSA increased during −6° (0.9 ± 0.4 cm^2^, *P* = 0.015), −12° (1.1 ± 0.5 cm^2^, *P* = 0.0001), and −18° HDT (1.3 ± 0.5 cm^2^, *P* < 0.0001), with the largest increase seen at −18°, compared with 0.6 ± 0.2 cm^2^ at supine baseline. On the arterial side, total CSA increased slightly during −18° HDT compared with supine baseline (0.8 ± 0.16 vs. 0.7 ± 0.1 cm^2^, *P* = 0.018). When the CSAs of the arteries were analyzed separately, arterial vasodilation occurred in the ICAs (0.27 ± 0.1 cm^2^ at −18° HDT and 0.2 ± 0.04 cm^2^ at baseline, *P* = 0.0003) and not the VAs (0.13 ± 0.04 cm^2^ at −18° HDT and 0.14 ± 0.05 cm^2^ at baseline, *P* = 0.099).

In addition, arterial and jugular venous blood flow velocities are shown in Fig. 3. Similar to the CSA, changes in arterial blood flow velocity were only seen in the ICA, decreasing during HDT from 22.4 ± 4.3 cm/s at 0° to 18.3 ± 6.13 cm/s at −6° (*P* = 0.01), 15.1 ± 4.9 cm/s at −12° (*P* < 0.0001), and 16.0 ± 5.0 cm/s at −18° HDT (*P* < 0.0001). VA velocity, however, did not change from baseline during HDT (*P* = 0.57). On the venous side, IJV velocity was found to decrease from 14.9 ± 7.1 cm/s at 0° to 8.1 ± 4.8 cm/s during −12° (*P* = 0.0004), and to 8.9 ± 6.6 cm/s during −18° (*P* = 0.002).

#### Arterial and venous blood flow.

Analysis of blood flow was performed in eight of nine subjects; in one subject, excessive motion artifact precluded analysis. Figure 2*B* illustrates the physiological response of arterial inflow and jugular venous outflow to HDT at the various angles. Total arterial inflow was found to significantly decrease from baseline during all HDT angles (*P* = 0.019, *P* < 0.0001, and *P* = 0.002 for −6°, −12°, and −18°, respectively) with a maximum decrease seen at −12° HDT with 606 ± 107 ml/min compared with 782 ± 93 ml/min at 0°. Further comparisons revealed a decrease in total arterial inflow from −6° to −12° HDT (*P* = 0.018); however, there was a trend toward increased flow from −12° to −18° HDT (*P* = 0.084). Total jugular venous outflow followed a similar pattern as arterial inflow during HDT (Fig. 2*B*), with a statistically significant decrease from 0° to −12° HDT (*P* = 0.039). In a similar manner to the arterial inflow, there was an increase in total jugular venous outflow from −12° to −18° HDT (405 ± 126 and 509 ± 104 ml/min, respectively, *P* < 0.01). The relative contribution of the ICAs vs. VAs to total arterial inflow did not vary across conditions (∼69 and 31%, respectively). On the venous side, there was a significant main effect of lateralization (*P* < 0.0001), with six of eight subjects having a higher mean flow through the right IJV during baseline and all HDT conditions. Figure 1 demonstrates one subject with a dominant left IJV.

Furthermore, although extra-jugular venous flow through the vertebral veins and other accessory pathways was not measured, it can be assumed to approximately equal the difference between measured arterial inflow and jugular outflow. The amount of venous outflow occurring through the jugular veins was found to be higher during the −12° HDT with 1% CO_2_ condition (84%) compared with 0°, −6°, −12°, and −18° HDT (68, 69, 67, and 75%, respectively).

#### Effects of 1% CO_2_ on arterial and venous systems.

The addition of a 1% CO_2_ atmosphere to −12° HDT induced an increase in arterial inflow compared with −12° with an ambient atmosphere (703 ± 115 vs. 606 ± 107 ml/min, *P* = 0.016, Fig. 4*A*). However, arterial inflow during −12° HDT with 1% CO_2_ was still significantly decreased compared with baseline (*P* = 0.016). Furthermore, −12° HDT (ambient atmosphere) decreased total jugular outflow (*P* = 0.039), and the addition of a 1% CO_2_ atmosphere during −12° HDT condition caused a significant increase in total jugular venous outflow, similar to the arterial side (*P* < 0.001) (Fig. 4*A*). The addition of 1% CO_2_ had no effect on arterial or jugular venous CSA compared with −12° with ambient atmosphere; however, the CSA was still increased compared with baseline (0.97 ± 0.4 vs. 0.62 ± 0.2 cm^2^, *P* = 0.006, Fig. 4*B*).

#### Cardiovascular variables.

Mean arterial blood pressure (MAP) increased from baseline during −6° (*P* < 0.0001), −12° (*P* = 0.008), and −18° HDT (*P* = 0.002), along with systolic blood pressure at −6° (*P* = 0.0008) and at −12° (*P* = 0.02), and diastolic blood pressure at −6° (*P* = 0.0002) and −18° HDT (*P* = 0.0007) (Table 1). However, there was no difference in MAP between the HDT conditions. Also, there was no change in HR from baseline during any condition (*P* = 0.944). Furthermore, there was no significant difference in the measured cardiovascular variables during −12° + 1% CO_2_ compared with baseline or −12° plus ambient air in any cardiovascular variable (Table 1).

**Table 1.** Various cardiovascular and respiratory variables at baseline and after 4- and 3.5-h head-down tilt

	Baseline (before −6°)	−6°	Baseline (before −12°)	−12°	Baseline (before −18°)	−18°	Baseline (before −12° +1% CO_2_)	−12° + 1% CO_2_
MAP, mmHg	73.17 ± 10.83	87.92 ± 11.18‡	75.3 ± 13.7	84.72 ± 14.32†	70.95 ± 7.16	82.13 ± 10.77†	76.85 ± 12.08	84.02 ± 12.22
SBP, mmHg	123.29 ± 17.37	140.98 ± 14.67‡	125.33 ± 9.94	138.15 ± 15.88*	124.67 ± 11.38	131.49 ± 20.28	134.38 ± 14.7	135.85 ± 15.98
DBP, mmHg	56.28 ± 9.07	68.71 ± 9.69‡	58.77 ± 13.44	66.06 ± 13.17	53.28 ± 6.81	64.99 ± 9.08‡	59.3 ± 11.38	66.5 ± 10.51
HR, beats/min	61.92 ± 9.7	60.8 ± 8.14	65.06 ± 8.85	63.31 ± 11.71	59.84 ± 8.23	58.01 ± 9.29	60.13 ± 9.61	60.41 ± 11.87
ET_CO_2__, %	5.14 ± 0.74	4.52 ± 0.56*	4.98 ± 0.96	4.76 ± 0.87	4.79 ± 0.97	4.67 ± 0.75	4.8 ± 0.68	5.23 ± 0.75
Minute ventilation, l/min	7.96 ± 3.12	8.17 ± 2.75	9.0 ± 3.02	9.51 ± 3.66	8.52 ± 3.97	8.8 ± 4.2	8.51 ± 3.98	8.17 ± 3.45

Values are means ± SD. All baseline measurements were taken in the supine position (0°).

MAP, mean arterial blood pressure; SBP, systolic arterial blood pressure; DBP, diastolic arterial blood pressure; HR, heart rate; ET_CO_2__, end-tidal carbon dioxide.

Difference from baseline:

* *P* < 0.05,

† *P* < 0.01,

‡ *P* < 0.001.

#### Respiratory variables.

ET_CO_2__ has been show to correlate well with changes in arterial partial pressure of CO_2_ (Pa_CO_2__) in subjects without pulmonary disease (6). Percentage of ET_CO_2__ was found to decrease slightly during −6° HDT compared with baseline (*P* = 0.02, Table 1); however, it did not change during any other condition. Compared with −12° HDT with ambient atmosphere, however, −12° HDT with a 1% CO_2_ atmosphere acted to increase ET_CO_2__ from 4.76 ± 0.87% during −12° HDT with ambient atmosphere to 5.23 ± 0.75% (*P* = 0.004). Minute ventilation was not found to change significantly from baseline during any condition (*P* = 0.43, Table 1).

## DISCUSSION

The main findings in our study are that HDT induced a decrease in arterial and venous blood flow as well as a large increase in IJV CSA. Furthermore, the addition of a 1% CO_2_ atmosphere during HDT led to an increase in blood flow on both the arterial and venous side compared with HDT with ambient atmosphere, bringing blood flow closer to supine baseline values.

Overall, progressive increases in IJV CSA were found with increasing hydrostatic pressure gradients induced by various angles of HDT, demonstrating the compliance of the venous system. Previously, Arbeille et al. (2) measured IJV CSA with ultrasound echography during −6° HDT and found an 8, 49, and 40% increase after 4–5, 7, and 42 days, respectively. Additionally, IJV CSA in microgravity was measured and found to increase 33–47% after 7 days of spaceflight and remained distended during 4–5 mo in space (2). Herault et al. (15) found increased IJV CSA by 23–30% after 1–5 mo in space. This level of microgravity-induced venous distension is closest to that found during −6° HDT in the present study; however, the measurements were taken with a different time scale and measurement technique. In another study, Arbeille et al. (3) measured the internal jugular and portal vein volumes and found increases in both after 15 days of spaceflight (178 and 36%, respectively) that was maintained during 5 mo of spaceflight, indicating blood pooling in the cephalad and pelvic regions. Jugular venous distension is a well-described finding in both HDT and microgravity (2, 12, 15) and could be due to unloading of tissue weight in the neck or increased venous pressure in the cephalad region. Petersen et al. (25) found an increase in ICP during −10° and −20° HDT, and, according to Davson's equation, ICP is directly dependent on cerebral venous pressure (9, 26). Therefore, HDT may lead to an increase in cerebral venous pressure due to the hydrostatic pressure gradient. Furthermore, there is no unloading of ICP in space, which normally occurs on Earth during positional changes, and could lead to structural changes in the eye associated with the VIIP syndrome (25).

In addition, total jugular venous outflow was found to decrease during HDT, showing a similar pattern as the arterial inflow. In the present study, total jugular venous outflow was found to be 534 ± 157 ml/min in the supine position, comparable with previously reported values (30, 36). Venous outflow through extra-jugular pathways (e.g., the vertebral veins) was not measured in this study; however, the IJVs are known to act as the main outflow pathway in the supine position (22). It is interesting to note that, in microgravity, there is no upright or supine position due to the lack of gravitationally induced hydrostatic pressure gradients. This study confirms that ∼70% of the venous outflow occurs through the jugular veins in the 0° HDT position (1, 5, 31), and that venous drainage balance was maintained at −6°, −12°, and −18° HDT. Interestingly, the portion of cerebral venous outflow through the jugular veins increased to ∼85% at −12° HDT with 1% CO_2_.

Furthermore, it was found that six of eight subjects in the presented study have higher flow in the right IJV than the left, comparable to previously reported values (30). The effect that venous lateralization has on the cerebral vascular system is not known; however, it is interesting to point out that the VIIP syndrome creates greater structural ophthalmic changes in the right eye vs. the left (21).

On the arterial side, the measured arterial inflow at baseline in the present study (782 ± 93 ml/min) is comparable with previously reported values for young subjects (7, 41, 43). Arterial inflow decreased during all angles of HDT compared with baseline; however, from −12° to −18° HDT, an unexpected increase in both arterial inflow (+9%) and venous outflow (+26%) was observed. Regulation of CBF is dependent on several interrelated physiological factors, including MAP and ICP [i.e., cerebral perfusion pressure (CPP)], cerebral autoregulation, cerebral metabolic demands (i.e., cerebral metabolic rate of oxygen consumption), partial pressures of O_2_ and CO_2_, and the autonomic nervous system (38). Specifically, in the HDT position, cerebral autoregulation may serve to limit the increase in CBF via myogenic vasoconstriction due to initial increases in CPP. Furthermore, cerebral autoregulation has been shown to be modified in the head-up tilt position by brief exposure to prior HDT and is dependent on the HDT angle (39). Here, we hypothesize that impeded intracranial venous return during HDT may increase postcapillary pressure, thus affecting the CPP gradient and blood flow in the cephalad regions, resulting in the demonstrated decrease in arterial blood flow. However, in addition to the hydrostatic effects, reduced blood flow may also lead to an accumulation of CBF-regulating metabolites (e.g., potassium) and CO_2_. Taken together, the accumulation of multiple local regulators may compete with the hydrostatic effects on decreasing CBF and explain the reversal in arterial blood flow seen between −12° and −18° HDT. There are several possible explanations why CBF unexpectedly increased at −18° compared with −12° HDT. First, it is possible that subjects had an increased cerebral metabolic rate of oxygen consumption due to anxiety or discomfort at this steeper angle; however, the subjects did not report any mental stress with the steeper HDT angles nor did pulse rate change. Furthermore, although we observed an increase in MAP during all HDT angles compared with supine, the largest difference was at the −6° rather than −18°. Second, we considered whether mechanical effects, such as shifting of abdominal contents toward the diaphragm, may have decreased minute ventilation and thus increased Pa_CO_2__ and CBF; however, no measureable change was found in minute ventilation during HDT compared with supine. Finally, there is a possibility that cerebral vasoconstrictive mechanisms reached a maximum and no longer attenuate the effects of increasing CPP at the steeper HDT angle. This phenomenon is similar to patients with hypertensive encephalopathy or eclampsia due to increases in MAP and thus CBF due to failure of cerebral autoregulatory mechanisms. However, the subjects in the present study were healthy individuals, and it has been previously shown that CPP does not change during −10° and −20° HDT (25).

Several studies have investigated CBF velocity during exposure to HDT and microgravity; however, results are inconsistent. Yasumasa et al. (40) and Kawai et al. (18) both found increased CBF velocity in the middle cerebral artery (MCA) during −6° HDT, whereas Sun et al. (32) observed a decrease during 21-day −6° HDT. Gelinas et al. (11) saw no change in CBF velocity in the MCA nor the posterior cerebral artery with −90° HDT, despite changes in MAP; however, ET_CO_2__ was maintained at baseline levels through respiratory coaching during HDT. In microgravity, both Bagian and Hackett (4), as well as Iwasaki et al. (17), found no change in MCA CBF velocity in astronauts after 10 h and 1–2 wk in microgravity, respectively. However, the aforementioned ground-based and spaceflight experiments utilized a different measurement method (transcranial Doppler), and, as CBF is a product of CBF velocity and vessel CSA, these reported changes in velocity may not necessarily reflect a change in flow. In addition, it has been found that cerebrovascular resistance is not solely modulated at the level of the arteriolar pial vessels, but rather the large extracranial vessels (ICA and VA) also contribute significantly to the total cerebrovascular resistance (10, 37). This unique role of the larger cerebral arteries in vascular resistance is thought to act as a protective mechanism for the microcirculation during variations in arterial blood pressure and to maintain constant blood flow to neuronal tissue. Furthermore, previous studies have shown that the ICA and VA are reactive to changes in arterial blood gases, with the ICA showing a 20% change in diameter through a Pa_CO_2__ range of 15–65 Torr (37). In the present study, the arterial CSA was found to increase during −18° HDT, but no change was seen during the hypercapnic HDT condition, possibly due to the relatively low level of ambient CO_2_.

Additionally, as with any closed environment, the CO_2_ levels on the ISS are significantly higher than terrestrial levels, typically ranging from 0.1 to 0.8% (20). CBF is known to be highly sensitive to changes in Pa_CO_2__, a potent vasodilator, and is, therefore, interesting to investigate the combined effects of HDT and increased ambient CO_2_. In the present study, the addition of a 1% CO_2_ atmosphere during −12° HDT increased both arterial inflow and jugular outflow (compared with −12° HDT with ambient atmosphere). However, enrichment of ambient air with CO_2_ does not directly translate to changes in ET_CO_2__, likely due to enhanced ventilatory dissipation of CO_2_. Imray et al. (16) found that supplementary 3% CO_2_ enrichment resulted in a 0.6% increase in ET_CO_2__, and the ET_CO_2__ response to 1% CO_2_ in this study amounted to ∼0.47% during −12° HDT. Previously, Henderson et al. (14) found increased heterogeneity of pulmonary blood flow after 1 h of −30° HDT, potentially due to increased pulmonary capillary pressure and fluid efflux in the lung. However, the present data suggest that HDT-related changes in ventilation/perfusion mismatch do not substantially hamper the ventilatory adjustments in CO_2_ dissipation in response to a 1% environmental CO_2_ challenge. While higher levels of ambient CO_2_ can increase sympathetic nervous outflow and induce a panic response, the relatively low level of CO_2_ exposure in this study is not expected to induce this response (8). Pa_CO_2__ and its influence on the regulation of CBF is defined as cerebral CO_2_ reactivity (28). Zuj et al. (44) found that long-duration spaceflight on the ISS impairs cerebrovascular autoregulation and CO_2_ reactivity in the MCA postflight, presumably due to chronic exposure to elevated ambient CO_2_. On Earth, Tymko et al. (35) found that body position (±90° and ±45° tilt) did not affect absolute cerebrovascular CO_2_ reactivity in the MCA or posterior cerebral artery (35).

Furthermore, as there is no natural convection in microgravity, astronauts may be exposed to pockets of very high concentrations of CO_2_ in areas of low ventilation (e.g., within sleeping quarters) in addition to the constantly elevated ambient CO_2_ level. A computational fluid dynamics analysis revealed that, without adequate ventilation, partial pressure of CO_2_ could rise above 9 Torr within 10 min around a sleeping astronaut's mouth and chin (29). Exposure to pockets of high CO_2_ concentrations could result in an onset of arterial vasodilatation, resulting in increased ICP, potentially having more pronounced effects in individuals with lower cranial compliance. In addition, individual CO_2_ sensitivity and retention may partially account for why some astronauts develop the VIIP syndrome, whereas others do not. However, individual variations could also be due to local fluctuations of CO_2_ that certain crew members may be more exposed to and warrants further investigation.

There are several limitations to consider when applying the results of our ground-based analog to spaceflight. Although HDT bed rest is an accepted model for simulated microgravity research (34), HDT does not eliminate the G_x_ gravitational influence on the body, but rather reverses the (G_z_) gravitational gradient to simulate a headward fluid shift, as occurs in microgravity. Therefore, this difference should be noted when extrapolating findings to real microgravity. Furthermore, during the HDT plus 1% CO_2_ condition, subjects inspired a predetermined breathing mixture as opposed to a computer-controlled system to continually alter arterial CO_2_ on an individual basis. This design was chosen to mimic the ISS environment by continuously exposing subjects to a low-level hypercapnic environment. However, as individual ventilatory responses to administered CO_2_ can be variable, subjects may have had differences in arterial and tissue partial pressure of CO_2_ compared with baseline and each other. It should also be noted that alterations in cerebral hemodynamics are just one hypothesized contributor to the VIIP syndrome; others include a pressure gradient between the intraocular pressure and ICP (anterior translaminar pressure gradient) (42), an enzymatic polymorphism (45), high-sodium diet, resistive exercise, anatomical shifting in microgravity, etc. (23).

In summary, this is the first study to measure PC-MRI-derived blood flow velocity, CSA, and absolute blood flow for cerebral arterial inflow and jugular venous outflow at various angles of HDT. It was found that HDT leads to signs of cerebral venous congestion, demonstrated by the large increase in CSA, and decreased blood flow, having implications for cerebral hemodynamics in microgravity, including contributing to the VIIP syndrome.

## GRANTS

This study was supported by the German Aerospace Center (identifier no. 2475 115). K. Marshall-Goebel was supported by a SpaceLife Scholarship of the Helmholtz Space Life Sciences Research School, which was funded by the Helmholtz Association (Helmholtz-Gemeinschaft, Grant no. VH-KO-300) for doctoral studies and received additional funds from the DLR, including the Aerospace Executive Board and the Institute of Aerospace Medicine. A. Eklund, J. Malm, and K. Ambarki were supported by a grant from the Rymdstyrelsen (Swedish National Space Board).

## DISCLOSURES

No conflicts of interest, financial or otherwise, are declared by the author(s).

## AUTHOR CONTRIBUTIONS

K.M.-G., K.A., A.E., J.M., E.M., and J.R. conception and design of research; K.M.-G., E.M., and D.G. performed experiments; K.M.-G., K.A., and E.B. analyzed data; K.M.-G., K.A., A.E., J.M., E.B., and J.R. interpreted results of experiments; K.M.-G. prepared figures; K.M.-G. drafted manuscript; K.M.-G., K.A., A.E., J.M., E.M., D.G., E.B., and J.R. edited and revised manuscript; K.M.-G., K.A., A.E., J.M., E.M., D.G., E.B., and J.R. approved final version of manuscript.
